# Infants’ Motor Proficiency and Statistical Learning for Actions

**DOI:** 10.3389/fpsyg.2017.02174

**Published:** 2017-12-12

**Authors:** Claire Monroy, Sarah Gerson, Sabine Hunnius

**Affiliations:** ^1^Donders Institute for Brain, Cognition and Behaviour, Radboud University Nijmegen, Nijmegen, Netherlands; ^2^Department of Otolaryngology – Head and Neck Surgery, Ohio State University Wexner Medical Center, Columbus, OH, United States; ^3^School of Psychology, Cardiff University, Cardiff, United Kingdom

**Keywords:** statistical learning, motor development, eye-tracking, infant cognition, action prediction

## Abstract

Prior research has shown that infants learn statistical regularities in action sequences better than they learn non-action event sequences. This is consistent with current theories claiming that the same mechanism guides action observation and action execution. The current eye-tracking study tested the prediction, based on these theories, that infants’ ability to learn statistical regularities in action sequences is modulated by their own motor abilities. Eight- to eleven-month-old infants observed an action sequence containing two deterministic action pairs (i.e., action *A* always followed by action *B*) embedded within an otherwise random sequence. One pair was performed with a whole-hand grasp. The second pair was performed with a pincer grasp, a fine motor skill that emerges around 9 months of age. Infants were then categorized into groups according to which grasp was dominant in their motor repertoire. Predictive looks to correct upcoming actions during the deterministic pairs were analyzed to measure whether infants learned and anticipated the sequence regularities. Findings indicate that infants learned the statistical regularities: across motor groups, they made more correct than incorrect predictive fixations to upcoming actions. Overall, learning was not significantly modulated by their dominant grasping abilities. However, infants with a dominant pincer grasp showed an earlier increase in correct predictions for the pincer grasp pair and not the whole-hand grasp. Likewise, infants with a dominant whole-hand grasp showed an early increase in correct predictions for the pair performed with a whole-hand grasp, and not the pincer grasp. Together, these findings suggest that infants’ ability to learn action sequences is facilitated when the observed action matches their own action repertoire. However, findings cannot be explained entirely by motor accounts, as infants also learned the actions less congruent with their own abilities. Findings are discussed in terms of the interplay between the motor system and additional non-motor resources during the acquisition of new motor skills in infancy.

## Introduction

Within the first months of life, infants begin to demonstrate remarkable abilities to form expectations about the actions they observe others perform. Infants readily anticipate the outcomes of observed actions and the trajectories of an actor’s movements as they unfold (Falck-Ytter et al., 2006; Ambrosini et al., 2013). For instance, they can predict that an adult will bring a cup to her mouth upon grasping it, long before they themselves can grasp mugs and drink from them (Hunnius and Bekkering, 2010). The mechanisms that support this ability have recently been a focus of intense study (for a review, see Hunnius and Bekkering, 2014). This body of work has centered around understanding how infants learn to anticipate observed actions based on observational experiences and their own developing action experiences.

Observational experiences create opportunities for infants to learn the statistical regularities in their environment. A recent surge of empirical work has provided convincing evidence that infants can detect multiple types of statistical regularities in different sensory domains from early in life (Aslin et al., 1998; Fiser and Aslin, 2001; Kirkham et al., 2002; Teinonen et al., 2009; Slone and Johnson, 2015). For instance, at 8 months of age, infants can segment novel auditory sequences into word-like units based on the transitional probabilities between syllables (Saffran et al., 1996). They can also form visual associations between objects and spatial locations based on their recurring co-occurrence (Kirkham et al., 2002) and can anticipate where an object will appear next based on those learned associations (Marcus et al., 1999).

Recent evidence has shown that statistical learning abilities extend to the action domain as well. Human action contains inherent sequential structure within a seemingly complex stream of motion (Baldwin and Baird, 2001; Zacks, 2004). From early in life, infants are sensitive to regularities in the actions they observe. For instance, 8-month-old infants segment observed action streams into separate units similarly to how they segment auditory sequences into words (Stahl et al., 2014). Within the first year of life, infants can learn to associate actions and the effects they produce, both for actions that they observe (Paulus et al., 2012) and those that they perform themselves (Verschoor et al., 2013). These findings add to a growing body of work which demonstrates that both observational and motor experiences contribute to infants’ emerging skills for processing and performing goal-directed actions.

Recently, researchers have begun to investigate whether, beyond segmentation, infants can also predict upcoming actions based on statistical learning. In a recent experiment, 18-month-old toddlers observed continuous action sequences with either deterministic or random transitional probabilities between actions. In a control condition, another group of toddlers observed the same sequence that featured self-propelled events rather than a human actor (Monroy et al., 2017b). Proportions of correct predictive looks preceding the deterministic actions increased over trials for the toddlers who observed the human actor, but not for those who observed the non-action visual events. These findings provided evidence that observing actions benefits action prediction above and beyond observing non-action perceptual sequences. One possible explanation is that prior motor experiences with the observed actions contributed to the enhanced learning, as these actions were all within the motor capabilities of toddlers.

Support for this hypothesis comes from a growing body of evidence illustrating that action prediction is tightly coupled to infants’ motor proficiency (Gredebäck and Kochukhova, 2010; Kanakogi and Itakura, 2011; Cannon et al., 2012; Gerson et al., 2015). For instance, in one recent experiment, infant and adult participants watched videos of other infants either crawling or walking across a room while their eye movements were recorded. Infants for whom crawling was their dominant form of locomotion predicted crawling more accurately than walking. In contrast, infants for whom walking was the dominant form of locomotion (and adults) were equally accurate at predicting both actions (Stapel et al., 2016). Training studies—in which infants are given novel experience with actions they have never yet performed—immediately alter how infants subsequently perceive those actions (Sommerville et al., 2005; Gerson and Woodward, 2014). Together, these findings suggest that infants process actions more efficiently once the actions are more strongly established in their own motor repertoire.

Current theories propose that activation of the motor system during action observation is the most likely mechanism underlying efficient action prediction (Flanagan and Johansson, 2003; Falck-Ytter et al., 2006). In line with this claim, findings from neuroimaging studies reveal that motor regions in the brain are activated when infants observe others’ actions. Activity in these regions is greater in response to actions with which infants have more motoric experience, and are therefore more dominant in their motor repertoire, relative to actions with which they have less or no motoric experience (Southgate et al., 2010; Gerson et al., 2015). In adults, motor activation is causally linked to predictive eye movements: introducing a competing motor task inhibits the ability to anticipate observed actions. Likewise, disrupting activity in the motor cortex via transcranial magnetic stimulation (TMS) impairs predictive eye movements, further suggesting that the motor system may even be necessary to successfully predict ongoing actions (Elsner et al., 2013).

Together, the current research shows that both observational statistical learning and action experience are central to infants’ action understanding (Hunnius and Bekkering, 2014). To date, a few studies have attempted to compare the relative contributions of self-produced and observed actions on infants’ action understanding (Gerson and Woodward, 2014) but none have examined how these two forms of experience might interact. We aimed to address this gap by asking whether newly acquired motor experience with grasping actions modulated infants’ abilities to learn statistical regularities between those same actions when they were viewed in continuous sequences. In other words, if infants recruit motor representations when they observe actions they can perform, does this help them to more easily detect the sequential regularities between those actions during observation?

To tackle this question, we exploited infants’ natural acquisition of a pincer grasp, a fine motor skill that emerges in the second half of the first year of life. Results from a prior study in our own lab indicated that the pincer grasp emerges between 8 and 11 months of age (Meyer et al., 2016). We thus expected 8- to 11-month-old infants to vary in the degree to which a whole hand (i.e., palmar) grasp and a pincer grasp were more dominant in their motor repertoire. In an eye-tracking experiment, infants were shown a video of an action sequence comprised of six possible object-directed actions. Within this sequence, there were two deterministic pairs in which one action always followed a second action with 100% probability and was followed by an effect. All other actions occurred in a random order. If infants learned the statistical structure of the action pairs, they should, in principle, make visual anticipations to the locations of the second action upon observing the first action (cf. Monroy et al., 2017b). Both actions of one pair were performed with a pincer grasp, whereas both actions of the second pair were performed with a whole-hand grasp. Following the video, a grasp test was conducted to assess each infant’s grasping proficiency.

We expected that, if infants learned the action pairs, they would make more visual anticipations toward the second action and/or its effect than to any other object during the first action of an action pair (i.e., a predictive time window). We also expected they would demonstrate an increase in correct visual anticipations to the second actions as the sequence progressed (Shafto et al., 2012; Monroy et al., 2017b). Finally, we hypothesized that infants would be better at learning the statistical regularities for the action pairs more dominant in their current motor repertoire. For example, we expected that those infants performing more whole-hand grasps, but not yet performing pincer grasp actions, would anticipate the second action of the whole hand pair more reliably than the pincer grasp pair. Infants for whom both actions are equally dominant in their motor repertoire should not show preferential learning for one pair over the other.

## Materials and Methods

### Participants

Forty-eight infants from 8 to 11 months of age were included in the final sample (**Table 1**). Infants were recruited from a database of interested families from the surrounding region who volunteered to participate. Seven additional infants were tested but excluded from the final sample due to calibration errors (*n* = 1) or failure to complete the observation phase due to excessive fussiness (*n* = 5). One infant made zero fixations on any of the trials of interest (i.e., the action pairs) and was also excluded from analyses. The study was approved by the ethical committee of behavioral science at the Faculty of Social Sciences in Nijmegen (Ethische Commissie Gedragswetenschappelijk Onderzoek; ECG2012-1301-006). All subjects gave written informed consent in accordance with the Declaration of Helsinki.

**Table 1:** Characteristics of the final sample.

Sample characteristics				Grasp test measures			
Motor group	*n*	Mean age in months (*SD*)	Gender (f:m)	Mean prop. pincer grasps (*SD*)	Mean prop. transitional grasps (*SD*)	Mean prop. hand grasps (*SD*)	Mean span in months^∗^ (*SD*)
Pincer-dominant	11	10.86 (*0.63*)	6:5	0.65 (*0.17*)	0.17 (*0.11*)	0.18 (*0.13*)	2.65 (*1.58*)
Transitional	22	10.07 (*0.94*)	6:16	0.21 (*0.16*)	0.45 (*0.30*)	0.35 (*0.25*)	1.93 (*1.10*)
Hand-dominant	15	9.94 (*0.97*)	7:8	0.06 (*0.08*)	0.13 (*0.11*)	0.80 (*0.10*)	1.65 (*0.90*)

*^∗^Mean span in months = number of months since infant first used a pincer grasp, per parent report (for descriptive purposes only; not used in analyses).*

### Stimuli

**FIGURE 1:**
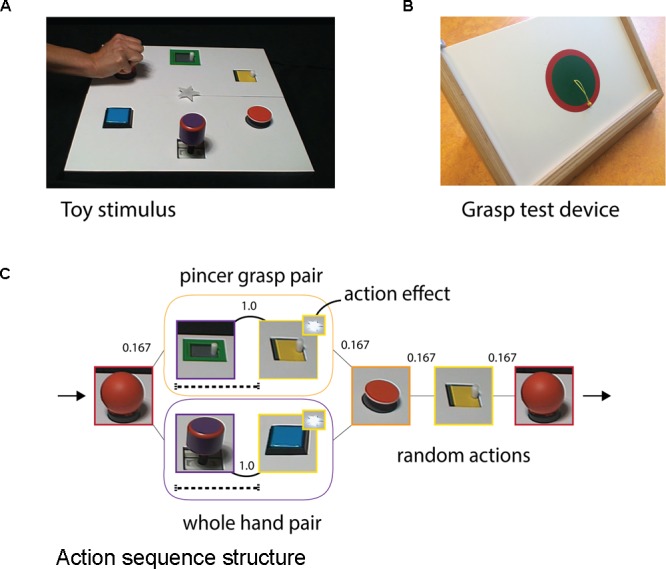
**(A)** Example frame from the video stimuli, in which an adult performed a continuous sequence of actions with the six possible objects on the toy. **(B)** Following observation, infants’ grasping abilities were assessed using the pictured apparatus, which required them to extract the bead from the wooden board. **(C)** Schematic illustrating the statistical structure of the action sequence containing two deterministic pairs that caused a light effect: one pair was performed with a pincer grasp and the second with a whole-hand grasp. Numbers represent transitional probabilities between paired and random actions. Dotted lines underneath the first action of a pair depict the 4 second period preceding the target actions in which predictive gaze fixations were analyzed.

Video stimuli were created featuring a toy with multiple objects that could be manipulated in distinct ways (**Figure 1A**). An adult actor performed a continuous action sequence with the various objects on the toy. For each action, the actor’s hand entered the screen nearest to the object upon which she would act, performed one action with that object (3 s), and then left the screen. This was followed by a brief pause (1 s) before the next action began. Only the actor’s hand was visible during each action.

Videos were divided into four blocks, with the viewing angle of the toy stimulus alternating between blocks to ensure that spatial location did not become a predictable cue. Attention-getter clips (4 s) were played between blocks followed by a still frame (1 s) of the toy (with no hand visible) to help the infant reorient to the new perspective. The entire sequence lasted approximately 7 minutes. Engaging background music accompanied the video stimuli and was unrelated to the stimulus presentation.

#### Action Sequence

The action sequence was structured as follows: two deterministic pairs were embedded within an otherwise pseudorandomized order of six object-directed actions. One pair was performed with a pincer grasp (Pincer pair) and consisted of the actions ‘slide’ followed by ‘open’; the second pair was performed with a whole-hand grasp (Hand pair) and consisted of ‘bend’ followed by ‘push’ (**Figure 1C**). The second actions of the pairs were labeled the *target* actions, as these were the actions that infants could learn to predict as they observed the unfolding sequence. Both pairs caused an action-effect, which was a green or a pink colored light in the center of the toy that turned on at the midpoint of the target actions. The light’s two colors (green and pink) always corresponded to the same pairs within one sequence. This matching was randomly counterbalanced across infants: one group always saw the Pincer pair activate a green light and the Hand pair activate a pink light, and the second group saw the reverse.

No action or pair occurred more than three times consecutively, and all elements (pairs and random actions) occurred with equal frequency. Target actions also occurred elsewhere in the sequence as random actions, to ensure that the effect only occurred after the two-step action pair and could not be independently associated with the target actions. Action sequences consisted of 96 total actions with 12 trials of each pair (Pincer and Hand).

#### Grasp Test Device

Infants’ grasping proficiency was assessed using a wooden apparatus (**Figure 1B**). A small and a large bead (3 and 5 mm diameter) were attached to strings that were threaded through removable wooden panels which fit into the apparatus frame.

### Procedure

The testing procedure consisted of an action observation phase followed by the grasp test. Infants were seated on a caretaker’s lap throughout both phases. During the action observation phase, eye movements were recorded continuously with a Tobii T120 eye-tracker (Tobii Technologies, Inc.) at 60 Hz. Gaze was calibrated using a standard nine-point calibration procedure until at least eight points were acquired or a maximum of three attempts. Immediately following calibration, infants were shown the video stimuli. Caretakers were requested to avert their gaze during calibration and to refrain from influencing their child during the observation phase.

After the sequence was completed (or until infants became too fussy to continue the observation task), caretakers and infants moved to a nearby table for the grasp test (adapted from the procedure of Meyer et al., 2016). The experimenter placed the test apparatus in front of the infant and performed a single demonstration of how to grasp the bead and pull it out. After returning the bead to its original position (**Figure 1B**), infants were given 1 minute to pull out each bead. Each time they removed the bead, the experimenter replaced it and the infant could try again. This phase was videotaped from a camera placed with full view of the infant for offline behavioral analysis. In addition, a parental questionnaire was administered prior to the testing session with questions about infants’ grasping history^1^.

## Data Analysis

### Eye-Tracking Data

Raw eye-tracking data was separated into discrete fixations using a custom software program (GSA; Philip van den Broek, Donders Institute) with a spatial filter of 30 pixels and a temporal filter of 100 ms. Regions of interest (ROIs) of equal size were defined around each object (i.e., action location) and around the action-effect (250 and 130 square pixels, respectively). Predictive time windows were defined as the 4 s from the first frame in which the hand appeared to perform the first action of a pair until the final frame just before the hand reappeared to perform the target (second) action (dashed bars; **Figure 1C**).

#### Calculation of Proportions of Predictive Fixations

Fixations to the target object and to the action-effect locations during predictive time windows were considered correct, whereas fixations to any other object were considered incorrect. Fixations to the location of ongoing actions were always excluded. We first calculated the proportions of correct (Eq. 1) and incorrect predictive fixations (Eq. 2) across all trials for each pair, divided the sum by the total fixations made to all ROIs. Total incorrect fixations were divided by four to yield the average number of fixations to an incorrect region; this measure has also been described elsewhere as an estimate of chance (Tummeltshammer and Kirkham, 2013). Proportions of correct and incorrect fixations were compared for each action pair, representing infants’ preference for anticipating a correct upcoming action and/or its effect relative to the other object locations. Proportions of correct fixations (Eq. 1) were also calculated per trial and analyzed using Generalized Estimating Equations (GEEs) to examine the emergence of predictive gaze over the course of the experiment. GEE analyses do not apply list-wise exclusion of cases and are thus advantageous for analyzing data with repeated measures that contain missing points, such as trials in which no anticipatory fixations occur (Zeger et al., 1988).

(1) $$\begin{array}{c} Correct=\frac{\#\mathrm{Fixations}\mathrm{to}\mathrm{target}\mathrm{and}\mathrm{effect}}{\mathrm{Total}\#\mathrm{fixations}\mathrm{to}\mathrm{all}\mathrm{ROIs}} \end{array}$$

(2) $$\begin{array}{c} Incorrect=\frac{\#\mathrm{Fixations}\mathrm{to}4non-targetobjects/4}{\mathrm{Total}\#\mathrm{fixations}\mathrm{to}\mathrm{all}\mathrm{ROIs}} \end{array}$$

Equations 1 and 2: Calculations of the proportion measures. ‘All ROIs’ refers to the six objects and the action-effect.

### Grasp Test

**FIGURE 2:**
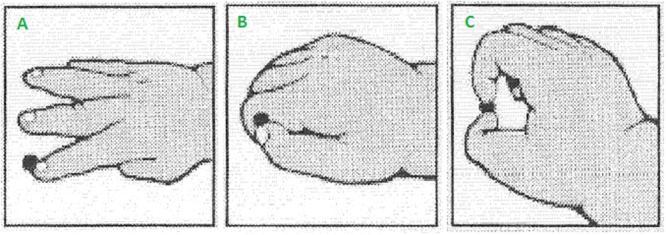
Illustrations depicting classification of grasping actions into whole hand **(A)**, transitional **(B)**, or pincer **(C)** grasps during the grasp test. Only **(C)** was considered a true pincer grasp as this action requires opposition of the thumb and forefinger. Image modified with permission from Erhardt (1994).

Infants’ ability to pull the beads out of the panels served as our measure of grasp proficiency (**Figure 2**). Video recordings of the grasp test phase were coded offline by a coder who was blind to the aims of the study. Each attempt to extract the bead from the device was coded as hand grasp, a transitional (i.e., inferior pincer) grasp, or a pincer grasp. Next, we calculated the proportion of times infants used each grasp type out of the total number of times he or she successfully extracted the beads, collapsed across small and large beads. Unsuccessful attempts were not coded.

Almost all infants could extract the bead from the device, while demonstrating different levels of grasping competence to do so. We thus classified infants according to the type of grasp they used most frequently to extract the bead, reasoning that this would reflect the motor ability most dominant in their current repertoire. Rather than acquiring new motor skills in sudden steps, infants’ motor skills emerge in a gradual, graded way (see Ambrosini et al., 2013 for another non-binary scoring method). Each infant was classified as *Pincer*-*dominant, Hand-dominant*, or *Transitional* depending on which grasp they used most (**Table 1**). For instance, if the relative proportions of an infant’s grasping actions were 0.58, 0.25, and 0.17 (pincer, transitional, and hand, respectively) this infant would be classified as a *Pincer*-*dominant* infant. Infants whose relative proportions were evenly distributed across grasp types—such as 0.33, 0.33, and 0.34—were also classified as *Transitional* infants. To avoid confusion, infant groups (*Pincer*-*dominant, Hand-dominant*, and *Transitional*) are presented in italics and action pairs (Pincer and Hand) in non-italics.

## Results

### Age Effects

A one-way ANOVA with age as dependent variable and Motor Group as a factor indicated that mean age differed significantly among the three motor groups, *F*(2,47) = 3.89, *p* = 0.03 (see **Table 1**). Pairwise comparisons revealed that the *Pincer-dominant* infants were significantly older than the *Hand-dominant* groups (mean difference [*MD*] = 27.96 days, *p* = 0.01) and the *Transitional* group (*MD* = 24.00 days; *p* = 0.02). There were no differences between the *Hand-dominant* and the *Transitional* infants (*MD* = 3.96, *p* = 0.66).

### Visual Attention

There were no differences between infant groups in overall looking time to all ROI throughout the entire video, or in the total number of fixations during predictive time windows (*p*s > 0.25). Thus, infants with different levels of motor experiences did not demonstrate different visual attention to the action sequence. Across groups, infants made anticipatory fixations to the target actions on 28.3% of the experimental trials for which gaze data was obtained across both pairs. This rate of anticipatory looks is typical for infants in this age range (Tummeltshammer and Kirkham, 2013).

To assess rates of anticipations over the course of the experiment, we conducted a linear, model-based General Estimating Equations (GEE) with an unstructured working correlation matrix. Each of the 12 trials from each pair (Pincer and Hand) was assigned a 1 if it included anticipation and a 0 if not. Trials were then collapsed into four time bins with three trials in each bin. Pair (Pincer and Hand) and Time Bin (T1, T2, T3, and T4) were entered as within-subjects repeated measures and Motor Group (*Pincer*-*dominant, Transitional*, and *Hand-dominant*) was entered as a between-subjects factor. Across all infants, rates of anticipation decreased significantly over the course of the experiment, χ^2^(3) = 51.14, *p* < 0.001. Pairwise comparisons revealed a consistent statistically significant decrease in the proportion of trials containing predictive fixations from each Time Bin to the next (e.g., from T1 to T2, from T2 to T3, and from T3 to T4). There were no other main effects or interactions (*p*s > 0.17), indicating that rates of visual attention did not differ across infants based on age or motor abilities.

### Correct vs. Incorrect

A one-sample Kolmogorov–Smirnov test revealed that proportion scores did not differ from a normal distribution for all dependent variables (*p*s > 0.05). A Levene’s test confirmed that the variances between the motor groups did not significantly differ from one another, *p* > 0.05.

To assess whether infants anticipated the next events in the sequence, we first compared proportions of correct fixations (Eq. 1) relative to incorrect fixations (Eq. 2) across the duration of the experiment. If infants learned the action pairs, proportions of fixations to correct locations should be higher than proportions to incorrect locations. In this analysis, the first trial was always excluded from calculations, as infants should not be able to correctly predict on the very first observation of each pair.

**FIGURE 3:**
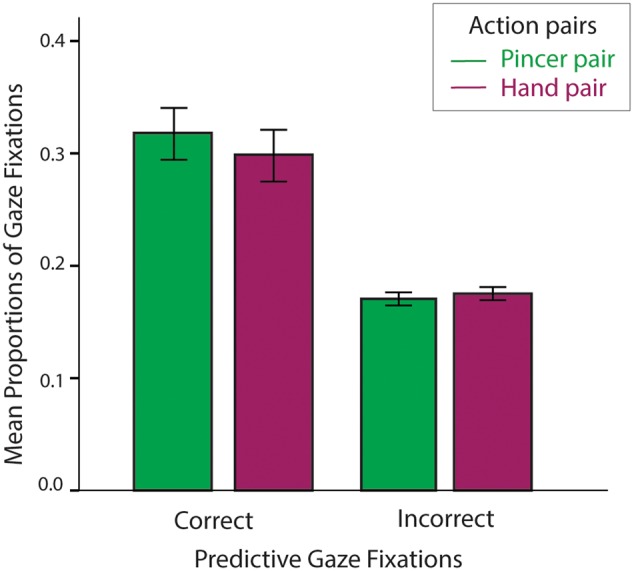
The mean proportions of correct and incorrect gaze fixations collapsed across motor groups. Bars represent standard errors of the mean.

We first compared correct and incorrect fixations across all infants to assess whether learning occurred at all. An ANOVA with Prediction (Correct vs. Incorrect) and Pair (Pincer and Hand) as within-subject factors and age as a covariate revealed a marginally significant main effect of Prediction, *F*(1,43) = 3.04, $\eta_{p}^{2}$ = 0.07, *p* = 0.09 and a significant effect of age, *p* = 0.04. Without age as a covariate, the main effect of Prediction was significant, *F*(1,46) = 22.08, $\eta_{p}^{2}$ = 0.32, *p* < 0.001 (**Figure 3**). Across pairs, correct proportions were higher than incorrect proportions (*MD* = 0.14, *SEM* = 0.03, *p* < 0.001)^2^. There was no main effect of pair (*p* = 0.64) nor was there an interaction between Pair and Prediction (*p* = 0.64).

We next added Motor Group (*Pincer-dominant, Transitional*, and *Hand-dominant*) as a between-subjects factor to assess whether correct and incorrect fixations varied among motor groups with age as a covariate. This yielded no main effects or interactions with Motor Group (*p*s > 0.60). Thus, as a group, infants selectively anticipated the correct action and its effects more frequently than they incorrectly anticipated other objects on the screen across all trials, and this did not significantly differ between pairs or motor groups.

### Learning Over Time

To further probe infants’ predictive gaze behaviors, we examined correct gaze proportions over time. We expected proportions of correct fixations (Eq.1) to increase over trials, as infants learned the sequence regularities. Correct fixations were entered into a linear, model-based GEE with an unstructured Working Correlation Matrix. Pair (Pincer and Hand) and Time Bin (T1, T2, T3, and T4) were entered as within-subjects repeated measures and Motor Group (*Pincer-dominant, Transitional*, and *Hand-dominant*) was entered as a between-subjects factor. Age was included as a covariate. The GEE revealed a significant main effect of Time Bin, χ^2^(3) = 31.00, *p* < 0.001, a significant interaction between Pair and Time Bin, χ^2^(3) = 15.047, *p* = 0.002, a significant interaction between Motor Group and Time Bin, χ^2^(6) = 23.33, *p* = 0.001, and a significant three-way interaction between Pair, Motor Group, and Time Bin, χ^2^(6) = 22.98, *p* = 0.001. There was no main effect of age, *p* = 0.16.

**FIGURE 4:**
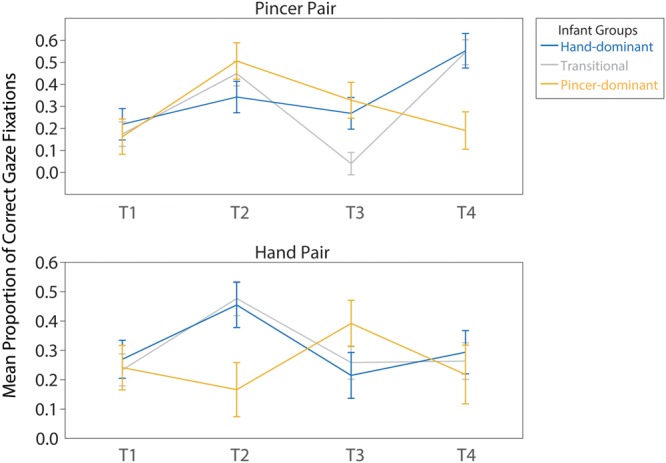
The mean proportion of correct gaze fixations over the four time bins of the experiment plotted separately for the Pincer pair (above) and the Hand pair (below). Lines represent the three infant motor groups. Bars represent standard errors.

To assess whether the time-course of learning differed among motor groups, pairwise comparisons were conducted to follow up on the significant three-way interaction between Pair, Motor Group, and Time Bin. This interaction effect is illustrated in **Figure 4**. Based on our *a priori* hypotheses, we expected the largest differences in predictive gaze between *Pincer*-*dominant* and *Hand-dominant* infants. Therefore, we first focus on the results from follow-up comparisons between these two groups, before turning to the results from the *Transitional* group.

For the *Pincer-dominant* motor group, correct predictions to the Pincer pair sharply increased from T1 to T2 (*MD* = 0.34, *SEM* = 0.12, *p* = 0.003) and then subsequently decreased from T2 to T4 (*MD* = 0.32, *SEM* = 0.10, *p* = 0.002). In contrast, the *Hand-dominant* group demonstrated no such increase in correct proportions from T1 to T2 (*MD* = 0.12, *SEM* = 0.10, *p* = 0.23). However, there were no differences between *Pincer-* and *Hand-dominant* groups for the Pincer pair at either T1 or T2 (*p*s > 0.14). It was not the case that the *Hand-dominant* infants showed no evidence for learning of the Pincer pair, as they did improve from T1 to T4 (*MD* = 0.33, *SEM* = 0.10, *p* = 0.001), but this increase was slower than that of the *Pincer*-*dominant* infants and did not emerge until the final quarter of the trials.

For the Hand pair, the pattern was reversed: the *Pincer*-*dominant* infants showed no difference in correct predictions from T1 to T2 (*MD* = 0.08, *SEM* = 0.12, *p* = 0.53) whereas correct predictions increased marginally for the *Hand-dominant* infants (*MD* = 0.19, *SEM* = 0.10, *p* = 0.07). There was no difference between *Pincer-dominant* and *Hand-dominant* groups for the Hand pair at T1 (*MD* = 0.03, *SEM* = 0.10, *p* = 0.78); however, they did differ significantly at T2 (*MD* = 0.29, *SEM* = 0.12, *p* = 0.017). The *Hand-dominant* infants subsequently showed a decrease in correct predictions from T2 to T3 (*MD* = 0.24, *SEM* = 0.10, *p* = 0.016) and no significant gain across the experiment (T1 to T4; *MD* = 0.02, *SEM* = 0.09, *p* = 0.80). The *Pincer*-*dominant* infants also showed no significant gain across the experiment for the Hand pair (*MD* = 0.02, *SEM* = 0.12, *p* = 0.85).

The *Transitional* group demonstrated a pattern in between that of the *Pincer*-*dominant* and the *Hand-dominant* group. For the Pincer pair, the transitional group also increased their correct fixations from T1 to T2 like the *Pincer*-*dominant* group (*MD* = 0.27, *SEM* = 0.08, *p* = 0.001), followed by a decrease from T2 to T3 (*MD* = -0.41, *SEM* = 0.08, *p* < 0.001). However, they again showed a second rise in correct predictions from T3 to T4 (*MD* = 0.51, *SEM* = 0.06, *p* < 0.001), like the *Hand-dominant* group, and overall their correct predictions increased from T1 to T4 (*MD* = 0.37, *SEM* = 0.08, *p* < 0.001). For the Hand pair, the transitional group closely followed the pattern of the *Hand-dominant* infants, with an initial gain in correct anticipations from T1 to T2 (*MD* = 0.24, *SEM* = 0.08, *p* = 0.003) followed by a decrease from T2 to T3 (*MD* = -0.22, *SEM* = 0.07, *p* = 0.002), and no significant change across the entire experiment (T1 to T4; *MD* = 0.03, *SEM* = 0.08, *p* = 0.70).

To sum up, the infants with more dominant pincer grasp abilities quickly detected the pair structure for actions performed with a pincer grasp and correctly anticipated the upcoming action or its effect within the first few observations. This was then followed by a decrease in correct predictions in later trials. Similarly, the infants with a dominant hand grasp showed a faster improvement in predictions for the actions performed with a hand grasp, followed by a decline in performance. The *Transitional* infants, whose motor experience fell between *Hand-dominant* and *Pincer-dominant* infants, showed fixation patterns which shared characteristics with both groups.

## Discussion

Observational statistical learning and motor experiences are both key pathways to infants’ developing action understanding and social-cognitive abilities (Hunnius and Bekkering, 2014). In the current eye-tracking experiment, we aimed to shed light on how these processes interact with one another during action observation. Infants observed an action sequence containing two deterministic pairs, one performed with a pincer grasp and the other with a whole-hand grasp. Predictive gaze to the second actions of each pair and their associated effects were measured as an indicator of statistical learning. Following observation, infants’ motor performance on a grasp test was used to determine their dominant grasp type. Our central hypothesis was that learning would be modulated by infants’ level of motor proficiency with the observed grasp type.

Findings revealed that infants, as a group, learned the transitional probabilities within the observed action sequences. Across pairs and motor groups, infants made more fixations to the correct upcoming actions and/or their effects than to other action locations on the screen. Consistent with prior findings with older infants (Monroy et al., 2017b), these results show that 8- to 11-month-olds can predict upcoming actions and their effects by learning transitional probabilities within an action sequence. Further, the general ability to predict upcoming actions was not driven by the specific motor action observed or by infants’ levels of motor proficiency.

A direct link between infants’ action perception and action production has previously been demonstrated for simple, isolated actions such as reaching and grasping (e.g., Gredebäck and Kochukhova, 2010; Kanakogi and Itakura, 2011). In the current experiment, we examined whether this link extends to situations in which infants need to use their statistical learning skills to predict upcoming sequential actions. This would be consistent with motor-based accounts of action understanding, which hypothesize that the motor system guides the generation of action predictions (Kilner et al., 2007).

Our data provide partial support for the notion that recent motor experiences influence infants’ statistical learning for action sequences. However, they do not provide conclusive support for the hypothesis that motor-based learning is essential for action predictions. Results showed that infants with a dominant pincer-hand grasp demonstrated an early increase in correct anticipations for the pincer grasp but not the hand grasp, indicating faster learning for the action dominant in their current motor repertoire. Likewise, infants with a dominant whole-hand grasp similarly demonstrated an early increase in correct anticipations for the hand grasp, but not the pincer grasp. The transitional infants—who likely had similar levels of proficiency with both actions—shared similar patterns with both groups. In sum, infants’ ability to learn action pairs based on statistical regularities was faster for the actions that are dominant in their current motor repertoire.

Faster learning for the action pair matching infants’ own motor abilities may reflect the influence of motor experiences on the ability to predict upcoming sequential actions, as we had hypothesized. According to motor-based accounts, the motor system combines prior knowledge with incoming sensory input to generate a prediction (Kilner et al., 2007). Motor experiences with the observed act are one important source of prior knowledge and allow the motor system to generate more precise predictions (Stapel et al., 2016). According to these views, the current data show that infants more readily predicted the actions for which they could recruit an established motor representation.

Unexpectedly, infants with a dominant whole-hand grasp, who had little experience performing a pincer grasp, still demonstrated learning for the pincer grasp pair. These findings suggest that the motor system was not the critical driving factor in infants’ action processing. However, there were three principal differences between the current experiment and the previous evidence for a closer link between motor skills and action prediction skills (e.g., Gredebäck and Kochukhova, 2010; Kanakogi and Itakura, 2011; Ambrosini et al., 2013). First, our paradigm featured sequential actions that differed in both statistical likelihood and type of grasp, whereas previous studies featured isolated reaching actions that differed only in the observed grasp. A recent study with adults revealed that, when action sequences contain varying degrees of predictability between actions, non-motor neural networks are activated that are traditionally involved in processing uncertainty within probabilistic perceptual input (Ahlheim et al., 2014). In a similar vein, perceptually difficult conditions engage additional brain regions beyond those typically activated during action observation (Lingnau and Petris, 2013). Thus, under uncertainty, domain-general regions outside the action-observation network become involved. One possibility is that additional non-motor processes became involved when infants’ own motor system does not have enough knowledge or sensory information to generate a precise prediction.

Secondly, prior research investigating anticipatory gaze and motor abilities have measured anticipations to the target, or end-point, of the actor’s reach-to-grasp actions. For instance, in Ambrosini et al. (2013), fixations during an actor’s reaching movement were recorded and anticipations were defined as any fixation to the object before the hand made contact. In this study, infants with faster anticipations to the object were considered more predictive than infants who anticipated later (see also Falck-Ytter et al., 2006; Gredebäck and Kochukhova, 2010; Kanakogi and Itakura, 2011; Rosander and von Hofsten, 2011). Some have interpreted such findings as evidence for the influence of an internal motor program, indicated by faster visual anticipations during the movement trajectory of observed action (i.e., a ‘gaze advantage,’ Rosander and von Hofsten, 2011). In the current experiment, we assessed anticipations to a future action step, rather than speed of gaze latencies during a reaching phase in which multiple motor cues are immediately available—such as movement velocity, hand shape and trajectory. Possibly, the difference between our findings and those of the aforementioned studies could indicate that motor experiences have a lesser impact on predicting the identity of an upcoming action step that cannot be predicted solely based on incoming motor information.

A third important difference between the current study and most prior research is our method of classifying motor ability. Here, we classified infants based on the relative dominance of each grasp type, rather than a binary classification of whether infants could in general perform the action or not (e.g., van Elk et al., 2008). This method more closely mirrors how infants’ motor development naturally unfolds (Ambrosini et al., 2013). Infants accumulate both visual and motor experiences with the fine-grained kinematics of an action—such as a certain muscle movement—before piecing together the entire action skill (Senna et al., 2016). Thus, although the whole-hand infants do not yet readily or voluntarily perform a pincer grasp, they may be able to take advantage of finer-grained motor cues for movements that they can do, such as the actor’s hand shape (Ambrosini et al., 2013). Indeed, some motor-based accounts claim that the motor system can predict even those actions well outside our own physical abilities—such as a bird’s flight—by approximating the link between the observed act and the motor system’s internal model (Schubotz, 2007).

Recent studies have further probed the influence of developing motor abilities on infants’ visual attention to actions and the objects and effects related to them. Importantly, shifts in visual attention may relate to the nature of what is attended to, rather than simply the overall amount. Though we observed no differences in global attention to objects between motor groups, there may have been differences in the microstructure of infants’ gaze shifts which could have led them to receive altered visual inputs according to the congruency between the observed action pair and their own motor expertise. For instance, Smith and Yu (2008) and Yu and Smith (2011) used microanalytic techniques to show that infants’ learning outcomes related to fine-grained patterns of gaze shifts between objects, and could be modeled by a simple associative learning model. Although in the current study we restricted our analyses to only predictive gaze, further analysis of the relations between fine-grained measures of visual attention and motor abilities would be an interesting avenue for future research and may shed additional light on the observed patterns.

Along these same lines, in the current study we considered predictions to upcoming actions and their effects as correct. We did not investigate whether infants only predicted the effect instead of the next upcoming action. Prior research has shown that infants of a similar age range rely on cues from action-effects to learn about sequential outcomes (Verschoor et al., 2010; Monroy et al., 2017a). It has also been suggested that the motor system predicts the effects of our own actions and those that we observe (Elsner et al., 2002). Thus, though it was not the focus of the current study, it would be interesting to further investigate whether the presence of action-effects might be an important aspect of the relation between motor experiences and statistical learning.

Surprisingly, following the initial rise in correct predictions for actions matching their own motor abilities, correct predictions subsequently declined. This decline was consistent across all infants and across action pairs, and showed that predictions did not follow a stable pattern over time. One simple explanation is a loss of attention to the stimuli. The proportions of trials containing predictive gaze fixations steadily decreased over the course of the experiment, indicating that infants made fewer predictions during later trials. Infants may have simply stopped anticipating after successfully making a few correct predictions, given that no new information was offered by subsequent repetitions of the action pairs. They may instead have begun to engage in other visual behaviors such as tracking the actor’s hands or exploring the visual scene in search of novel information. Some paradigms use so-called ‘occluders’ to encourage participants to make visual anticipations (e.g., Hunt and Aslin, 2001; Johnson et al., 2003; Falck-Ytter et al., 2006; Paulus et al., 2011). In contrast, in our study, all objects were freely visible throughout the entire demonstration which, though more ecologically valid, may also have ‘discouraged’ anticipatory gaze. The conditions under which infants reliably and consistently anticipate actions, particularly in naturalistic, live contexts, are an important avenue for future research which we are currently pursuing.

## Conclusion

Given the accumulating evidence for the role of the motor system in facilitating action processing, can motor accounts explain the current findings? We propose that infants were engaging their motor systems as they processed the action sequence: when the observed action pairs were congruent with the grasping action most dominant in their motor repertoire, infants demonstrated a rapid increase in correct predictions. However, differences between motor groups were subtle. Learning was not tightly constrained by infants’ level of motor expertise, suggesting that additional cognitive processes come into play when infants need to use their statistical learning skills to generate action predictions. These findings further demonstrate that infants’ action prediction abilities cannot solely be explained by motor accounts, but likely reflect the recruitment of both motor and non-motor strategies when prediction requires learning statistical regularities in action sequences.

## Author Contributions

CM led the writing of this manuscript. CM, SG, and SH designed the experimental paradigm. CM collected the data and conducted the analyses. SG and SH both provided supervision and discussion regarding the analyses. SG and SH reviewed and contributed edits to this manuscript.

## Conflict of Interest Statement

The authors declare that the research was conducted in the absence of any commercial or financial relationships that could be construed as a potential conflict of interest.

## Footnotes

1 This was collected as a secondary measure to support the data from the grasp test and was not used in the current analyses.

2 Due to the uneven and relatively small sizes of our participant groups and relatively small samples, we repeated this analysis using a non-parametric Wilcoxon Signed Ranks test to compare Correct and Incorrect fixations across pairs and motor groups. This analysis confirmed that the mean proportion of Correct fixations was significantly greater than the proportion of Incorrect fixations, *z* = -3.91, *p* < 0.001, *r* = -0.56.
